# Lesion-Level Association Between Artificial Intelligence–Derived Coronary Calcium Volume and Plaque Vulnerability

**DOI:** 10.1016/j.jacadv.2026.103077

**Published:** 2026-07-28

**Authors:** Ruben G.A. van der Waerden, Joske L. van der Zande, Jan-Quinten Mol, Pierandrea Cancian, Thijs J. Luttikholt, Xiaojin Gu, Leah Heil, Tomasz Roleder, Jos Thannhauser, Simone Saitta, Clara I. Sánchez, Bram van Ginneken, Ivana Išgum, Niels van Royen, Rick H.J.A. Volleberg

**Affiliations:** a Department of Cardiology, Radboud University Medical Center, Nijmegen, the Netherlands; b Diagnostic Image Analysis Group, Radboud University Medical Center, Nijmegen, the Netherlands; c Department of Biomedical Engineering and Physics, Amsterdam University Medical Center, Amsterdam, the Netherlands; d Informatics Institute, University of Amsterdam, Amsterdam, the Netherlands; e Amsterdam Cardiovascular Sciences, Amsterdam UMC, Amsterdam, the Netherlands; f Faculty of Medicine, Wrocław University of Science and Technology, Wrocław, Poland; g Department of Radiology and Nuclear Medicine, Amsterdam University Medical Center University of Amsterdam, Amsterdam, the Netherlands; h Department of Radiology, Mayo Clinic, Rochester, Minnesota, USA

**Keywords:** artificial intelligence, calcium volume, coronary calcification, optical coherence tomography, vulnerable plaque

## Abstract

**Background:**

Extensively calcified lesions are typically at low risk for lesion-level acute events, which may be related to less vulnerable plaques.

**Objectives:**

This study sought to evaluate the lesion-level association between total calcification volume and plaque vulnerability.

**Methods:**

Data were derived from the prospective, observational PECTUS-obs study, in which patients with myocardial infarction underwent intracoronary optical coherence tomography (OCT) imaging of all fractional flow reserve-negative nonculprit lesions. OCT images were analyzed by a core laboratory for plaque vulnerability features (lipid-rich plaque, thin-cap fibroatheroma, layered plaque, macrophage accumulation, microvessel, and cholesterol crystal) and by a validated artificial intelligence algorithm (OCT-AID) for automated volumetric calcium quantification. Total calcium volume was indexed for lesion length and stratified by quartiles (Q1-Q4), from lowest to highest.

**Results:**

Among 488 lesions from 414 patients, calcium plaques were identified in 470 (96.3%) lesions. The lesion-level median indexed total calcium volume ranged from 0.004 mm^3^/mm (IQR: 0.001-0.011) in Q1 to 0.83 mm^3^/mm (IQR: 0.63-1.18) in Q4. The lower quartiles demonstrated the highest prevalence of lipid-rich plaques, macrophage accumulation, and microvessels. Thin-cap fibroatheroma and layered plaques accumulated predominantly in the middle quartiles (Q2-Q3). Cholesterol crystals showed no significant differences between quartiles. Overall, Q1-Q3 lesions demonstrated a greater mean number of plaque vulnerability features than Q4 lesions (*P* < 0.001 for all).

**Conclusions:**

Most severely calcified nonculprit lesions demonstrate a significantly lower prevalence of plaque vulnerability features as compared to less calcified, more lipid-rich lesions, which may contribute to the lower lesion-level acute event rate observed in heavily calcified lesions.

Coronary artery calcification is a key marker of atherosclerosis, providing insights into plaque stability and cardiovascular risk.^1^ Although extensive and dense calcifications, typically evaluated using coronary computed tomography and expressed in the Agatston calcium score, are indicative of high atherosclerotic burden, they are generally at low risk for acute lesion-level events.^2^, ^3^, ^4^, ^5^, ^6^ This disparity between patient-level risk and lesion-level acute events may be related to a lower prevalence of plaque vulnerability features in extensively calcified lesions. Early stage calcification, characterized by small calcium volumes with limited longitudinal and circumferential extent, has been associated with plaque inflammation and vulnerability.^1^^,^^7^ During natural plaque progression, lesions can undergo advanced calcification, which may result in a noninflammatory plaque phenotype with low plaque vulnerability and lower risk of acute events. However, little in vivo data exists supporting this hypothesis. In particular, nonculprit lesions, from which many recurrent events originate,^3^ remain underexplored.

Intracoronary optical coherence tomography (OCT) enables detailed assessment of plaque vulnerability and calcium volumes across the full range from small deposits to large, extensively calcified plaques.^8^ However, previous studies relied on manual measurements of the maximum circumferential and longitudinal extent of calcification,^9^, ^10^, ^11^, ^12^ which may not provide a complete appreciation of the total calcium volume. With recent advances, artificial intelligence (AI) enables automated and comprehensive volumetric analysis of OCT, overcoming the limitations of manual measurements of singular frames,^13^ and allowing detailed evaluation of the association between calcium volume and plaque vulnerability features in a 3-dimensional (3D) fashion. This study therefore aimed to evaluate this association in vivo in nonculprit lesions of patients with recent myocardial infarction.

## Methods

### Study design

This was a post hoc analysis of the prospective, multicenter PECTUS-obs study (NCT03857971), which evaluated the association between OCT-identified high-risk plaques in fractional flow reserve-negative nonculprit lesions and major adverse cardiovascular events in patients with recent myocardial infarction. The design and its primary findings were published previously.^14^^,^^15^ In brief, patients were eligible for inclusion if, after treatment of the infarct-related artery, invasive coronary angiography identified at least 1 nonculprit lesion of intermediate stenosis (visually estimated diameter stenosis 30% to 90%) in any coronary artery that was not hemodynamically significant (fractional flow reserve >0.80), which is referred to as a target lesion. Patients subsequently underwent OCT imaging of all eligible target lesions using the Dragonfly Optis imaging catheter (Abbott Vascular) under fluoroscopic guidance to ensure the OCT imaging could be co-registered to specific artery segments. The study was conducted in accordance with the 1964 Declaration of Helsinki and was approved by the institutional review board and/or medical ethics committee of each participating center. Written informed consent was obtained from all participants. For this analysis, all patients with at least 1 analyzable OCT pullback were included.

### Manual OCT assessment of plaque vulnerability

OCT pullbacks were manually assessed by an independent OCT core laboratory, using the angiogram as a reference for lesion localization. At the lesion level, images were analyzed for the minimum lumen area, lesion length, and percentage area stenosis, as well as for the presence of plaque vulnerability features, including lipid-rich plaque, thin-cap fibroatheroma (TCFA), layered plaque, macrophage accumulation, microvessel, and cholesterol crystal. All features were assessed in accordance with consensus guidelines.^5^^,^^16^ Lipid-rich plaque was defined as a signal-poor region with diffuse borders and high signal attenuation, exhibiting a lipid arc ≥90°. TCFA was defined as a lipid-rich plaque with a fibrous cap thickness <65 μm. Layered plaque was defined as plaque structure exhibiting one or more distinct layers of differing optical densities, clearly separated from underlying tissue. Macrophage accumulation was identified as punctate high-intensity regions with strong backscattering and signal attenuation. Microvessels were defined as small, sharply delineated low-intensity regions visible in at least 3 consecutive frames. Cholesterol crystals were characterized by thin, linear structures with high intensity. The total number of plaque vulnerability features was defined as the sum of the individual plaque vulnerability features.

### Automated calcium evaluation

#### Full-vessel segmentation

OCT images were automatically segmented using OCT-AID, a deep learning-based semantic segmentation model developed and validated by our group.^17^ For this study, the latest version of OCT-AID, built upon the nnU-net-v2 framework, was utilized.^18^^,^^19^ Detailed methodology and training procedures, including the training/test data split, were described previously.^20^ This model performs pixelwise segmentation of each OCT frame, distinguishing among 10 classes: background, guidewire artifact, lumen, intima, media, lipid, calcium, side branch, plaque rupture, and thrombus. Calcium segmentations generated by the model were used for all calcification-related measurements.

#### Postprocessing

OCT-AID predictions were postprocessed using frame-level connected component analysis with 4-connectivity to remove small groups of isolated predicted pixels.^17^ Only pixels connected along the horizontal or vertical axis were grouped. Diagonally connected pixels were considered as separate components. For calcium predictions, areas smaller than 100 pixels (≈0.01 mm^2^) were reclassified to the largest neighboring class.

#### Exclusion of attenuation artifacts

Frames with severe attenuation artifacts, primarily due to residual blood, were systematically identified using a previously developed deep learning-based artifact detection model.^21^ An OCT frame was excluded from further analysis if severe attenuation artifacts were detected in more than 25% of the A-lines in the respective frame. Lesions were subsequently excluded if over 50% of the frames within a lesion were excluded.

#### 3D calcium representation

Segmentation predictions were transformed from catheter-centered to lumen-centered coordinates to align calcified predictions across consecutive frames and enable consistent 3D reconstruction. To enhance consistency in the 3D calcium prediction, single frames without calcium predictions were retrospectively corrected if they were flanked by calcium-positive frames. In such cases, only pixels spatially overlapping with calcium regions from both adjacent frames were reclassified as calcium. Likewise, when a single isolated frame was excluded based on artifacts, the overlapping calcium region from adjacent artifact-free frames was interpolated to fill in the affected area. If 2 or more consecutive frames were identified as affected by artifacts, these frames were excluded entirely from the calcium quantification analysis.

Calcifications were then grouped based on 3D spatial connectivity. Noncontiguous calcified regions were considered distinct calcifications.

### Quantitative calcification analysis

For each calcium plaque, the following features were calculated: volume (mm^3^), length (mm), maximum arc (°), minimum depth (μm, defined as the shortest distance between the lumen and adluminal border of calcium), and maximum thickness (μm, defined as the longest distance between the adluminal and abluminal border of calcium). Total calcium volume (mm^3^) per lesion was computed by summing the volumes of all individual calcium plaques within that lesion, and total indexed calcium volume was defined as total calcium volume divided by the lesion length (mm^3^/mm). Lesions without any calcium were assigned a total calcium volume of zero and included in the lowest quartile (Q1). For lesion-level analyses, all lesions were subsequently grouped in quartiles (Q1-Q4) according to the total indexed calcium volume, from lowest to highest.

### Statistical analysis

Categorical variables are reported as counts (percentages), and continuous variables are presented as mean ± SD for normally distributed variables or median (IQR) for non-normally distributed variables.

Baseline characteristics and calcification-level measures across the total population were evaluated descriptively. Lesion-level calcification measures and baseline variables per total indexed calcium volume quartile were evaluated using generalized estimating equations (GEE) with an exchangeable correlation structure to account for repeated measures within the same patient. The inferential target of all GEE models in the study was the marginal (population-averaged) association across patients. An exchangeable working correlation structure was selected because lesions within the same patient were expected to exhibit similar intra patient correlation considering the systemic nature of atherosclerosis. Categorical variables were evaluated with logit link, and continuous outcomes were evaluated using an identity link, except for variables with a gamma distribution, which were modeled using a log link. Differences between quartiles were primarily evaluated using the Wald chi-square test and subsequent pairwise comparisons were performed using Tukey honestly significant difference test for baseline characteristics. Trends across quartiles were evaluated by modeling quartiles as ordinal variable within the same GEE framework and testing the linear trend using Wald chi-square statistics. Binary comparisons between Q1–Q3 and Q4 were derived from GEE models using a binary group variable, with *P* values obtained using Wald chi-square test.

Prevalence estimates and 95% CIs were obtained from GEE binomial models with a logit link. The association between the total indexed calcium volume per quartile and individual plaque vulnerability features and the total number of plaque vulnerability features was similarly assessed using the Wald chi-square test with group-wise comparisons followed by pairwise comparison. Model-based marginal means (95% CI) for the total number of plaque vulnerability features were obtained from GEE models.

Subgroup analyses were performed in patients with vs without lipid-lowering therapy (LLT) and sensitivity analyses were performed by: 1) stratifying lesions into quartiles based on calcium arc; and 2) excluding lesions without any calcium.

Receiver operating characteristic (ROC) curve analyses were performed to evaluate the ability of OCT-derived calcium measures to discriminate vulnerable plaque features. The area under the curve (AUC) of different calcium measures was compared using a cluster bootstrap (2,000 iterations, patient-level resampling with replacement) to account for clustering of lesions within patients.

Statistical analyses were conducted using IBM SPSS Statistics (version 27.0.1.0; IBM Corp). Correction for multiple testing was not applied considering the exploratory nature of these analyses. A *P* value of <0.05 was considered statistically significant.

## Results

### Baseline characteristics

Among 438 patients included in PECTUS-obs, 420 patients (494 lesions) were evaluated by the core laboratory. After excluding 6 patients with no AI-analyzable lesion, 414 patients (488 lesions) were included in further analyses (Supplemental Figure 1).

Baseline characteristics are presented in Table 1. The mean population age was 63 ± 10 years, and 79 (19.1%) patients were females. Diabetes and hypertension were present in 14.5% and 52.9% of patients, respectively. In total, 63 (15.2%) patients had a prior myocardial infarction, with 125 (30.5%) patients having a positive family history of premature atherosclerosis. In addition, 213 (51.4%) patients had presented with ST-segment elevation myocardial infarction and 108 (26.1%) patients were using LLT.

**Table 1:** Baseline Characteristics (N = 414)

Age (y)	63 ± 10
Female	79 (19.1%)
BMI (kg/m^2^)	27.8 ± 4.6
Smoking	
Current smoking	122 (29.7%)
Previous smoking	125 (30.4%)
Hypertension	219 (52.9%)
Type 1 or 2 diabetes	60 (14.5%)
Hypercholesterolemia	152 (36.8%)
Family history of premature atherosclerosis	125 (30.5%)
Previous MI	63 (15.2%)
Previous PCI	62 (15.0%)
Previous CVA	8 (1.9%)
History of carotid artery disease	13 (3.1%)
History of PAD	17 (4.1%)
STEMI presentation	213 (51.4%)
Cholesterol levels	
Total (mmol/L)	5.0 ± 1.4
LDL (mmol/L)	3.0 ± 1.2
Triglyceride level (mmol/L)	1.6 (1.1-2.4)
eGFR (mL/min/1.73 m^2^)	79.8 ± 19.1
CRP (mg/L)	2.9 (1.0-5.0)
Leukocyte count (×10^9^/L)	9.8 ± 3.2
Lipid-lowering therapy at presentation	108 (26.1%)

Values are n (%), mean ± SD, or median (IQR).

BMI = body mass index (calculated as weight in kilograms divided by height in meters squared); CRP = C-reactive protein; CVA = cerebrovascular accident; eGFR = estimated glomerular filtration rate; LDL = low-density lipoprotein; MI = myocardial infarction; PAD = peripheral artery disease; PCI = percutaneous coronary intervention; STEMI = ST-segment elevation myocardial infarction.

### Calcification identification and quantification

A total of 7,656 calcium plaques were automatically detected. The median calcium volume was 0.014 mm^3^ (IQR: 0.004-0.100 mm^3^), median calcium length was 0.2 mm (IQR: 0.1-0.6 mm), median maximum calcium arc was 19° (IQR: 11°-36°), median minimum calcium depth was 179 μm (108-294 μm), and median maximum calcium thickness was 242 μm (IQR: 141-466 μm).

### Lesion-level characteristics per quartile

**Figure 1:**
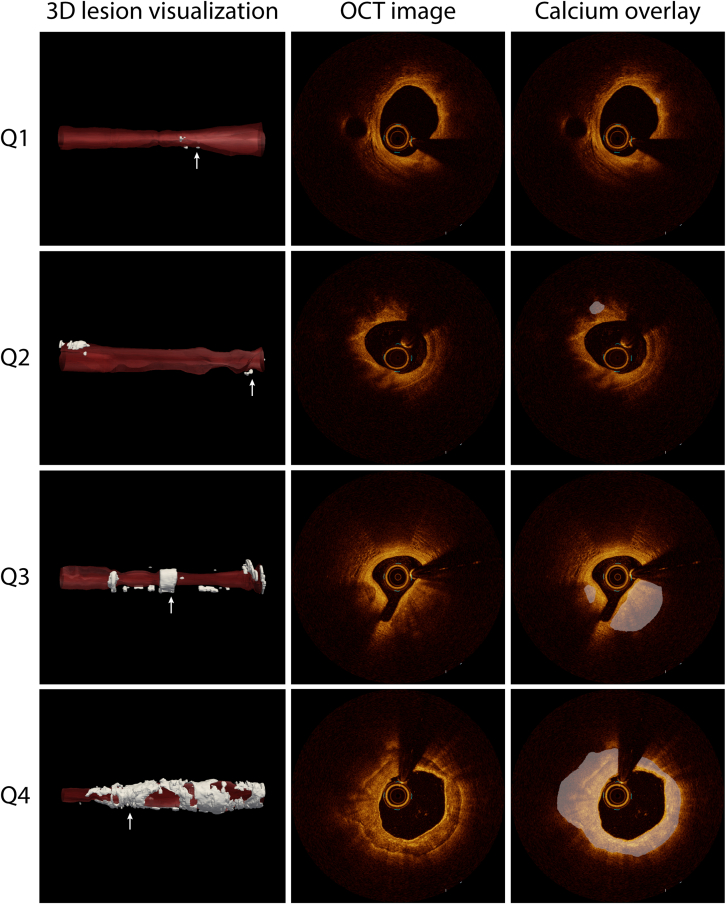
Automated Calcium Segmentation First column: 3D visualization of lesions, with lumen (red) and calcium (white) predictions per quartile (Q1-Q4), with a white arrow indicating the cross-sectional OCT location. Second column: cross-sectional OCT image. Third column: corresponding calcium overlay predictions in white. OCT = optical coherence tomography

An overview of lesion-level characteristics, stratified in quartiles by total indexed calcium volume, is provided in Table 2, with lesion-level baseline characteristics per quartile detailed in Supplemental Table 1. The median total indexed calcium volume increased across quartiles, from 0.004 mm^3^/mm (IQR: 0.001-0.011 mm^3^/mm) in Q1, to 0.06 mm^3^/mm (IQR: 0.04-0.10 mm^3^/mm) in Q2, 0.25 mm^3^/mm (IQR: 0.19-0.32 mm^3^/mm) in Q3, and 0.83 mm^3^/mm (IQR: 0.63-1.18 mm^3^/mm) in Q4 (*P*_trend_ < 0.001) (Figure 1, Supplemental Figure 2). Similarly, the number of calcium plaques per lesion increased gradually from 4 (IQR: 1-7) in Q1 to 19 (IQR: 12-37) in Q4 (*P*_trend_ < 0.001), with 18 lesions (4%) without any calcium in Q1.

**Table 2:** OCT-Defined Calcium Characteristics at Lesion Level

	Q1 (n = 122)	Q2 (n = 122)	Q3 (n = 122)	Q4 (n = 122)	*P*_trend_
Quantitative lesion characterization					
Lesion length (mm)^a^	15.7 ± 7.8	18.0 ± 8.2	20.7 ± 9.4	20.7 ± 9.0	<0.001
Minimum lumen area (mm^2^)^a^^,^^b^	2.17 (1.44-2.92)	2.55 (1.79-3.62)	2.26 (1.63-3.27)	2.41 (1.75-3.42)	0.86
Area stenosis (%)^a^	61.8 ± 16.9	61.8 ± 16.1	62.9 ± 15.6	62.8 ± 15.8	0.57
Max. plaque burden (%)^c^	61.9 ± 10.6	63.9 ± 9.0	67.1 ± 8.3	65.4 ± 9.4	<0.001
Max. lipid arc (°)^c^	218 ± 87	249 ± 84	255 ± 76	256 ± 82	<0.001
Calcium quantification^c^					
Total indexed calcium volume (mm^3^/mm)	0.004 (0.001-0.011)	0.06 (0.04-0.10)	0.25 (0.19-0.32)	0.83 (0.63-1.18)	<0.001
Number of calcium plaques	4 (1-7)	11 (6-17)	19 (11-27)	19 (12-33)	<0.001
Calcium length (mm)^d^	0.30 (0.20-0.60)	1.40 (0.98-2.00)	3.00 (2.10-4.20)	6.36 (4.40-9.20)	<0.001
Calcium volume (mm^3^)^d^	0.04 (0.01-0.11)	0.56 (0.30-0.86)	2.28 (1.46-3.37)	8.29 (4.80-14.93)	<0.001
Calcium arc (°)^d^	28 (18-42)	54 (38-70)	82 (63-108)	140 (94-201)	<0.001
Calcium depth (mm)^d^	198 (108-275)	155 (90-213)	162 (94-227)	138 (84-205)	0.004
Calcium thickness (mm)^d^	343 (205-412)	681 (543-812)	940 (821-1,051)	1,178 (1,025-1,317)	<0.001
Calcium score^d^^,^^e^	0 (0-0)	1 (1-1)	1 (1-1)	2 (2-4)	<0.001

Values are mean ± SD or median (IQR).

a Core laboratory-derived.

b *Modeled using a gamma distribution*.

c Artificial intelligence–derived.

d Calcium characteristic are derived from the calcification with the largest volume in each lesion; only lesions with any calcium prediction were included (n = 470).

e Calcium score based on the “rule of 5”: calcium arc >180° (2 points), calcium thickness >0.5 mm (1 point), and calcium length >5 mm (1 point); total score ranges from 0 to 4.

### Association between plaque vulnerability features and calcium volume index

Lipid-rich plaques were identified in 363 (74.4%; 95% CI: 70.2%-78.1%) lesions, with an inverse relation between total indexed calcium volume and the prevalence of lipid-rich plaques. The prevalence of lipid-rich plaques was significantly higher in Q1 (89.3%; 95% CI: 82.3%-93.7%) than in Q3 (78.7%; 95% CI: 70.7%-84.9%, *P* = 0.022) and in Q4 (48.4%; 95% CI: 39.5%-57.3%, *P* < 0.001) with a similar numerical lower prevalence in Q2 (81.1%; 95% CI: 73.3%-87.2%, *P* = 0.08). The prevalence in Q4 was also significantly lower than in Q2 and Q3 (*P* < 0.001 for both).

TCFA was present in 134 (27.5%; 95% CI: 23.5%-31.5%) lesions and was more prevalent in Q2 (32.8%; 95% CI: 24.7%-41.2%) and Q3 (36.9%; 95% CI: 28.8%-45.8%) compared with Q1 (21.3%; 95% CI: 14.4%-29.5%, *P* = 0.047 and *P* = 0.007, respectively) and Q4 (18.9%; 95% CI: 12.4%-26.9%, *P* = 0.014 and *P* = 0.001, respectively). Layered plaques were observed in 100 (20.5%; 95% CI: 16.9%-24.1%) lesions and were more prevalent in Q1 (18.9%; 95% CI: 12.8%-26.7%), Q2 (24.6%; 95% CI: 17.7%-33.0%) and Q3 (28.7%; 95% CI: 21.0%-37.3%) than in Q4 (9.8%; 95% CI: 5.7%-16.5%; *P* = 0.045, *P* = 0.002 and *P* < 0.001, respectively). Despite a numerical higher prevalence in Q2 and Q3, no statistically significant difference was observed between Q1 and Q2 and Q3 (*P* = 0.28 and *P* = 0.07, respectively).

Macrophage accumulation was found in 112 (23.0%; 95% CI: 19.4%-26.9%) lesions, and was significantly higher in Q1 (33.6%; 95% CI: 25.8%-42.4%), Q2 (22.1%; 95% CI: 15.6%-30.3%), and Q3 (27.9%; 95% CI: 20.6%-36.6%) than in Q4 (8.2%; 95% CI: 4.5%-14.3%, *P* < 0.001, *P* = 0.001, and *P* < 0.001, respectively). Microvessels were present in 78 (16.0%; 95% CI: 13.0%-19.5%) lesions, with higher prevalence in Q1 (20.5%; 95% CI: 14.0%-29.1%), Q2 (18.9%; 95% CI: 12.9%-26.%7), and Q3 (17.2%; 95% CI: 11.3%-25.4%) compared with Q4 (7.4%; 95% CI: 3.9%-13.6%; *P* = 0.004, *P* = 0.007, and *P* = 0.024, respectively).

**Figure 2:**
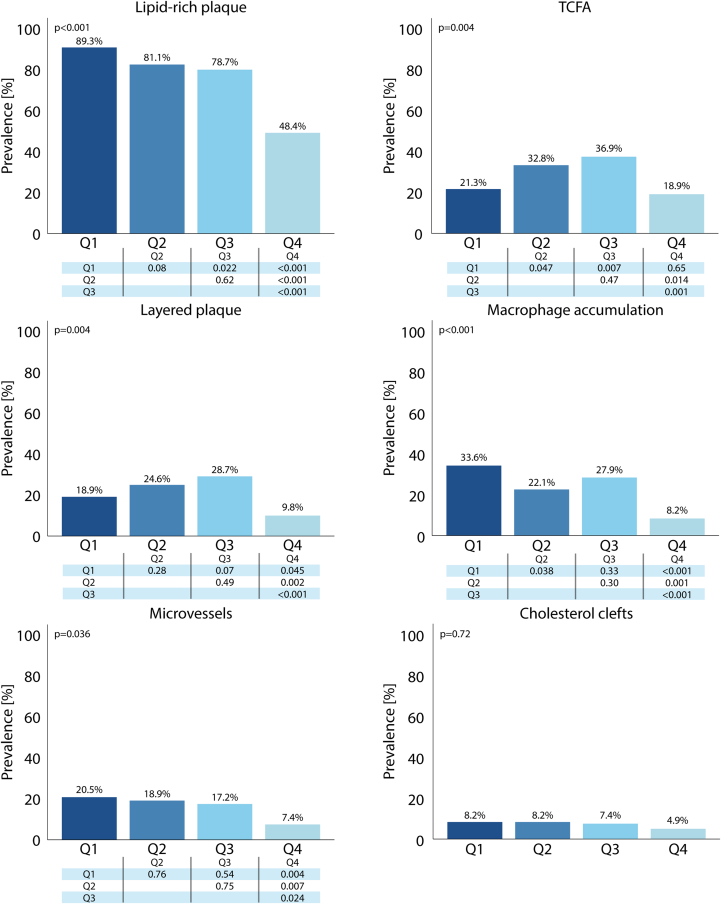
Individual Lesion-Level Vulnerability Features and Total Indexed Calcium Volume Prevalence of individual plaque vulnerability features across indexed calcium volume quartiles. Pairwise comparison *P* values between indexed calcium volume quartiles for individual plaque vulnerability feature are shown in the accompanying table only when a significant across-group difference is present. TCFA = thin-cap fibroatheroma.

**Figure 3:**
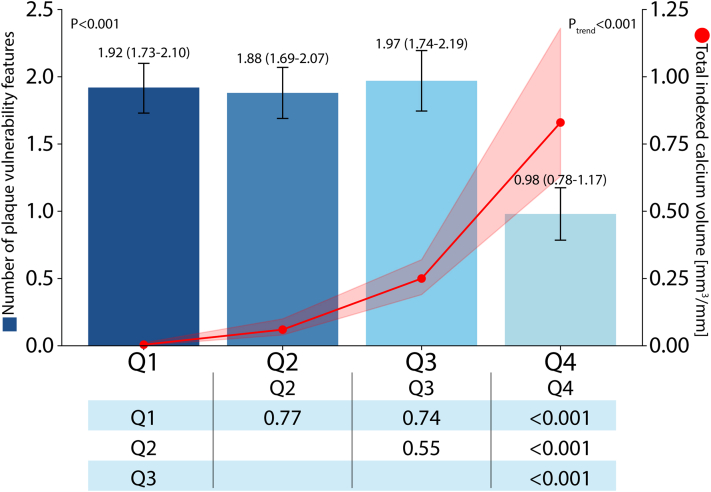
Association Between Plaque Vulnerability and Total Indexed Calcium Volume The marginal mean (95% CI) number of plaque vulnerability features vs total calcium volume index per lesion, arranged left to right by increasing total index calcium volume quartiles. Pairwise comparison *P* values between indexed calcium volume quartiles for number of plaque vulnerability features are shown in the accompanying table.

Cholesterol crystals were observed in 35 (7.2%; 95% CI: 5.2%-9.8%) lesions, with no significant differences between quartiles. The associations between the individual plaque vulnerability features and total indexed calcium volume are summarized in Figure 2. The mean total number of plaque vulnerability features was lower in Q4 as compared to Q1, Q2, and Q3 (*P* < 0.001 for all) (Figure 3). The association between calcium volume and plaque vulnerability features was comparable in patients with LLT and without LLT (Supplemental Figures 3 and 4). Binary comparison of Q4 vs Q1-Q3 (Supplemental Table 2) and sensitivity analyses using quartile-based stratification by calcium arc demonstrated consistent results (Supplemental Figures 5 and 6), and similar findings were observed when analyses were restricted to calcium-positive lesions (Supplemental Figures 7 and 8). Effect size estimates from the GEE models, expressed as OR with 95% CIs for each plaque vulnerability feature across calcium volume quartiles, are provided in Supplemental Table 3.

**Figure 4:**
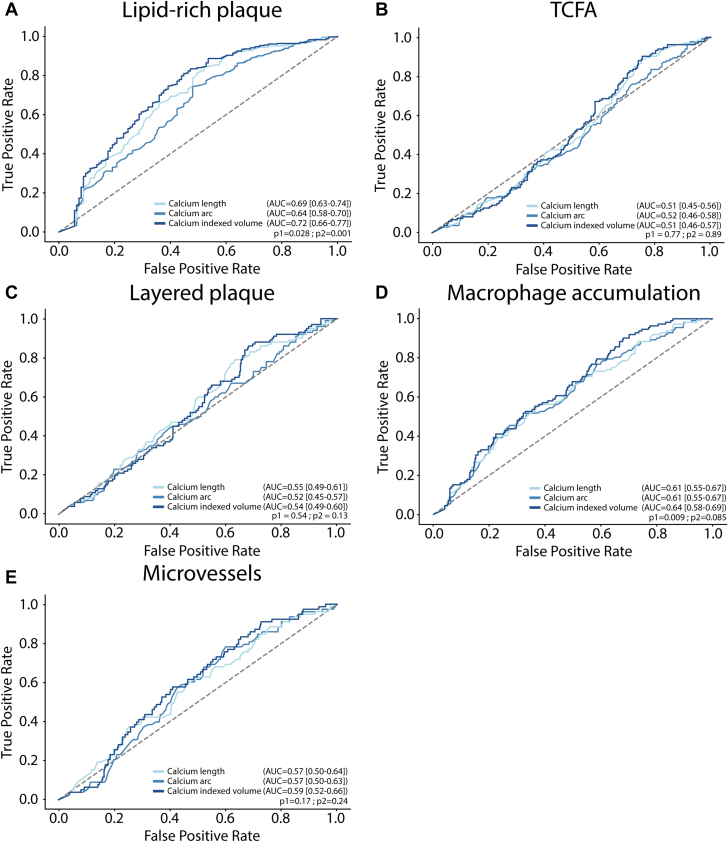
Receiver-Operating Characteristics Curves of Calcium Metrics With Plaque Vulnerability Receiver-operating characteristic curves of calcium length, arc, and volume index for discriminating (A) lipid-rich plaque, (B) TCFA, (C) layered plaque, (D) macrophages, and (E) microvessels. *P* values are reported for comparisons between total indexed calcium volume and calcium length (p1) and total indexed calcium volume and calcium arc (p2). AUC = area under the curve; other abbreviation as in Figure 2.

A reduction in total indexed calcium volume demonstrated fair discriminative value for the presence of lipid-rich plaques (AUC: 0.72; 95% CI: 0.66-0.77), and poor discriminative value for macrophage accumulation (AUC: 0.64; 95% CI: 0.59-0.69) and microvessels (AUC: 0.59; 95% CI: 0.53-0.65) (Figure 4). The discriminative value was significantly higher for the total indexed calcium volume than for the calcium length (*P* = 0.022) or arc (*P* < 0.001) for lipid-rich, and higher than calcium length for macrophage accumulation (*P* = 0.010). ROC analyses showed no significant discriminatory value for TCFA or layered plaque.

**Central Illustration:**
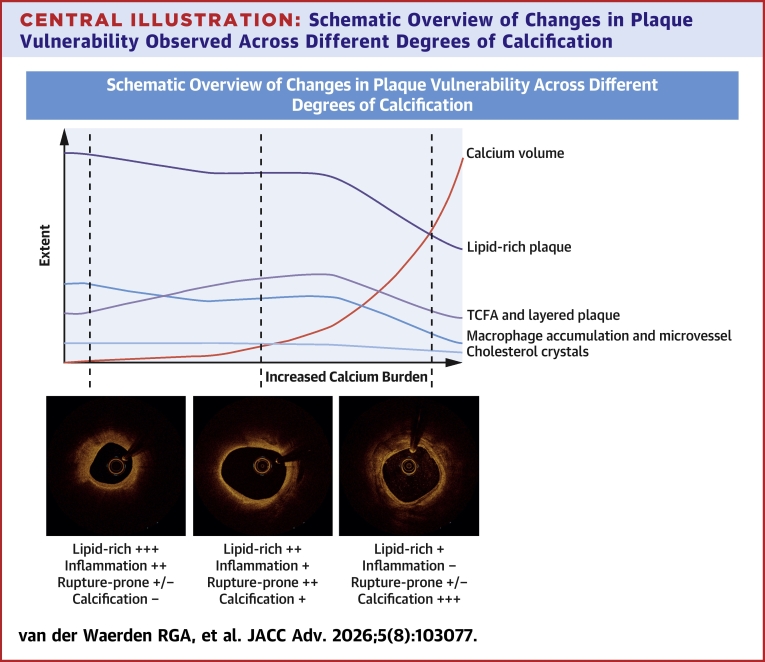
Schematic Overview of Changes in Plaque Vulnerability Observed Across Different Degrees of Calcification Lesions with no to little calcium are characterized by lipid-rich plaque, macrophage accumulation and microvessels. Intermediate calcified plaques are characterized by a rupture-prone status with a higher prevalence of TCFA and layered plaques. Most extensively calcified plaques demonstrate minimal vulnerable plaque characteristics. TCFA = thin-cap fibroatheroma.

## Discussion

This study evaluated the lesion-level association between AI-derived total calcium volume from OCT and plaque vulnerability features in nonflow limiting nonculprit lesions of patients with recent myocardial infarction, with several key findings: 1) several individual and the total number of plaque vulnerability features, including lipid-rich plaques, TCFA, layered plaque, macrophage accumulation, and microvessels, were the least prevalent in most extensively calcified lesions; 2) TCFA and layered plaque appeared to accumulate in the middle quartiles; and 3) total indexed calcium volume demonstrated a better discriminative ability than calcium length and arc for lipid-rich plaques. These observations indicate that plaque vulnerability may be a dynamic process with initially a large lipid pool (Q1), followed by a phase of progressive calcification with accumulating vulnerable plaque characteristics like TCFA and layered plaques (Q2-Q3) and finally extensive calcification with disappearance of vulnerable characteristics (Central Illustration). Leveraging AI-based volumetric calcium quantification, this study provides invasive evidence corroborating the long-standing histopathological concept that the low rate of acute lesion-level events originating from severely calcified lesions^6^ is related to a lower prevalence of rupture-prone vulnerable plaques.

This study suggests that coronary calcifications may follow a dynamic biological continuum. Early, lipid-rich plaques (Q1) are initially free of calcification and subsequently accumulate microcalcifications, predominantly arising from smooth muscle cell apoptosis or being generated in macrophage-derived matrix vesicles.^1^ Small, early-stage calcifications have been associated with increased local and systemic inflammation,^11^^,^^12^ which also induces fibrous cap thinning, transitioning lesions into a vulnerable phenotype.^22^ Correspondingly, we observed the highest prevalence of macrophage accumulation in the lower quartiles.

As lesions progress through intermediate stages (Q2-Q3), mixed vulnerable plaques emerge, with accumulating TCFA and layered plaque, whereas inflammation activity gradually decreases. This observation corresponds with recent studies that similarly found no linear relationship between culprit lesion calcification and OCT-identified TCFA and layered plaque.^10^^,^^23^ However, direct study-to-study comparison are challenged by differences in calcium quantification methods, and selected patient populations and lesion subtypes. Irrespectively, these findings suggest that particularly those intermediately calcified lesions may be the most rupture-prone, which corresponds with a recent histopathological study demonstrating that most ruptured plaques in patients with sudden cardiac death demonstrated some degree of calcification, predominantly mild to moderate.^24^ The copresence of TCFA and layered plaque further underscores the rupture-prone status of these lesions. From a biomechanical point of view, microcalcifications alter intraplaque stress predisposing lesions to fibrous cap thinning and rupture.^25^ In addition, fibrous cap thinning may not occur immediately after plaque formation but may be resultant of sustained local and systemic inflammation, providing a hypothesis behind the lower prevalence of TCFA in the lowest quartile. The null finding for cholesterol crystals should be interpreted cautiously considering its low prevalence and associated lack of statistical power.

Over time, microcalcifications coalesce into macrocalcifications redistributing mechanical stress, which may be accelerated through LLT.^26^ By then, local inflammation is reduced, with fewer macrophage accumulations and microvessels, and the plaque phenotype transitions from a rupture-prone phase to a stable phenotype, arguably making these lesions at lower risk for acute lesion-level events. Intriguingly, the lower observed prevalence of plaque vulnerability features in the upper quartile was consistent in patients with vs without LLT, indicating that extensive calcification results in stable plaque phenotypes both following natural plaque progression and LLT-induced plaque progression.

Overall, these observations elaborate earlier imaging studies. In PROSPECT II, using near-infrared spectroscopy-intravascular ultrasound, nonculprit events were significantly associated with high lipid content and large plaque burden.^3^ In the SCOT-HEART trial, using coronary computed tomography angiography, high-risk features such as low-attenuation plaque and positive remodeling predicted acute coronary syndromes.^27^ Similarly, the TACTICS registry, using OCT, demonstrated that lipid-rich plaques are prone to rupture, and extensively calcified plaques (max. calcium arc ≥180°) are rarely observed among ruptured lesions.^28^ Our study uniquely evaluated the association between total calcium volume and plaque vulnerability in nonculprit lesions after myocardial infarction. Leveraging AI-based OCT image interpretation, we volumetrically quantified calcium from OCT, contrasting traditional OCT-based assessment of calcium reliant on manual, selective measurement of length and arc. Automated volumetric quantification enables a more complete and precise evaluation of the total calcium burden across the entire lesion. We also report a better discriminatory value of total indexed calcium volume than of calcium length or calcium arc for the identification of lipid-rich plaque and macrophage accumulation, indicating the potential of AI-driven volumetric analysis to provide a more comprehensive understanding of the relationship between calcification and plaque vulnerability. By bridging histopathologic theory with in vivo lesion-level imaging, this AI-based volumetric calcium assessment offers a novel and biologically plausible framework for understanding plaque stabilization. The lack of significant discriminatory value for TCFA and layered plaque reflects the non-monotonic relationship between total indexed calcium volume and these phenotypes, which limits the interpretability of ROC-based analyses.

From a clinical perspective, indexed calcium volume may help contextualize plaque vulnerability during diagnostic coronary angiography. A higher indexed calcium burden may reflect more advanced atherosclerotic remodeling and indicate stable lesions that may be at low risk for acute coronary event related to plaque disruption. However, precursors of eruptive calcified nodules, which contribute to a smaller number of acute coronary events, are less well established.^29^

### Study Limitations

First, calcium characteristics were derived from AI-based segmentation with automated frame-to-frame interpolation and without manual adaptations, which may result in some misclassifications. However, the segmentation model used in this study has high performance for calcified plaque evaluation.^20^ Second, the study represents a cross-sectional imaging study without serial imaging and the present analysis was not predefined. The results therefore remain exploratory and cannot be interpreted as definitive proof of the hypothesized longitudinal plaque progression. Third, the results only apply to nonculprit lesions in patients with recent myocardial infarction and may not be directly generalizable to other patient populations. Fourth, the modest number of lesion-level events in the PECTUS-obs study^14^ precluded meaningful evaluation of the relationship between calcium volume and clinical outcomes. Fifth, in heavily calcified plaques, OCT shadowing may limit visualization of deeper plaque components which could contribute to the lower detection of vulnerability features. Finally, the impact of human interobserver variability in the evaluation of plaque vulnerability could not be evaluated.

## Conclusion

Most severely calcified nonculprit lesions demonstrate a significantly lower prevalence of plaque vulnerability as compared to lesions containing no to little calcium, providing a potential explanation for the lower rate of acute lesion-level events originating from extensively calcified lesions compared to lesions containing less calcium.

## Funding support and author disclosures

This publication is part of the project ROBUST: Trustworthy AI-based Systems for Sustainable Growth with project number KICH3.LTP.20.006, which is (partly) financed by the Dutch Research Council (NWO), Abbott, and the Dutch Ministry of Economic Affairs and Climate Policy (EZK) under the program LTP KIC 2020-2023. Funding partners were not involved in the collection, analysis and interpretation of the data, and in the preparation, review, or approval of the manuscript. PECTUS-obs was financially supported by 10.13039/100000046Abbott and Health∼Holland (grant number LSHM19102). Funding parties were not involved in the design and conduct of the study; collection, management, analysis, and interpretation of the data; preparation, review and approval of the manuscript; and decision to submit the manuscript for publication. Dr Sanchez has received institutional grants from 10.13039/100000046Abbott. Dr van Ginneken is shareholder from Thirona and Plain Medical. Dr Išgum has received institutional grants from the Dutch Science Foundation, 10.13039/100000046Abbott, Pie Medical Imaging BV, Philips, and Esaote SpA. Dr van Royen has received institutional research grants from 10.13039/100000046Abbott, 10.13039/100004320Philips, 10.13039/501100005035Biotronik, and 10.13039/100004374Medtronic. All other authors have reported that they have no relationships relevant to the contents of this paper to disclose.
