# Supplementary material for: Lesion-Level Association Between Artificial Intelligence–Derived Coronary Calcium Volume and Plaque Vulnerability
Source: JACC Adv. 2026 Jul 28;5(8):103077. doi: 10.1016/j.jacadv.2026.103077 (PMC13430182; doi:10.1016/j.jacadv.2026.103077)
Supplement: Supplemental Material [file mmc1.docx]

Supplemental material

Content

[Supplemental Figures 3](#_Toc233197761)

[Supplemental Figure 1 Study flow diagram 3](#_Toc233197762)

[Supplemental Figure 2 Total indexed calcium volume 4](#_Toc233197763)

[Supplemental Figure 3 Individual lesion-level vulnerability features and total indexed calcium volume, stratified by lipid-lowering therapy 5](#_Toc233197764)

[Supplemental Figure 4 Association between plaque vulnerability and total indexed calcium volume, stratified by lipid-lowering therapy 7](#_Toc233197765)

[Supplemental Figure 5 Individual lesion-level vulnerability features and calcium arc 8](#_Toc233197766)

[Supplemental Figure 6 Association between plaque vulnerability and calcium arc 9](#_Toc233197767)

[Supplemental Figure 7 Individual lesion‑level vulnerability features and total indexed calcium volume (calcium‑positive lesions only) 10](#_Toc233197768)

[Supplemental Figure 8 Association between plaque vulnerability and total indexed calcium volume (calcium‑positive lesions only) 12](#_Toc233197769)

[Supplemental Tables 13](#_Toc233197770)

[Supplemental Table 1 Lesion-level baseline characteristics per quartile 13](#_Toc233197771)

[Supplemental Table 2 Binary comparison (Q1–Q3 vs Q4) of lesion-level vulnerability features 15](#_Toc233197772)

[Supplemental Table 3 Effect sizes for plaque vulnerability features across calcium volume quartiles 16](#_Toc233197773)

# Supplemental Figures

## Supplemental Figure 1 Study flow diagram

*
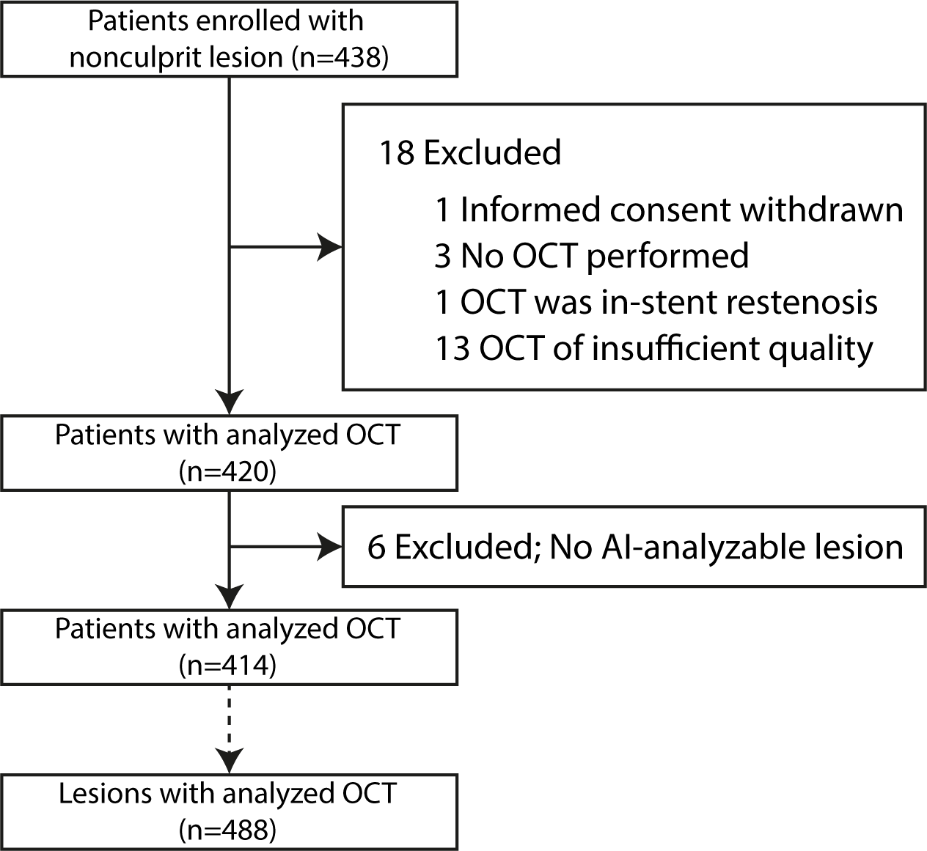
*

*Study flow for patient selection, exclusions, and AI calcium analysis.*

OCT, Optical coherence tomography.

## Supplemental Figure 2 Total indexed calcium volume


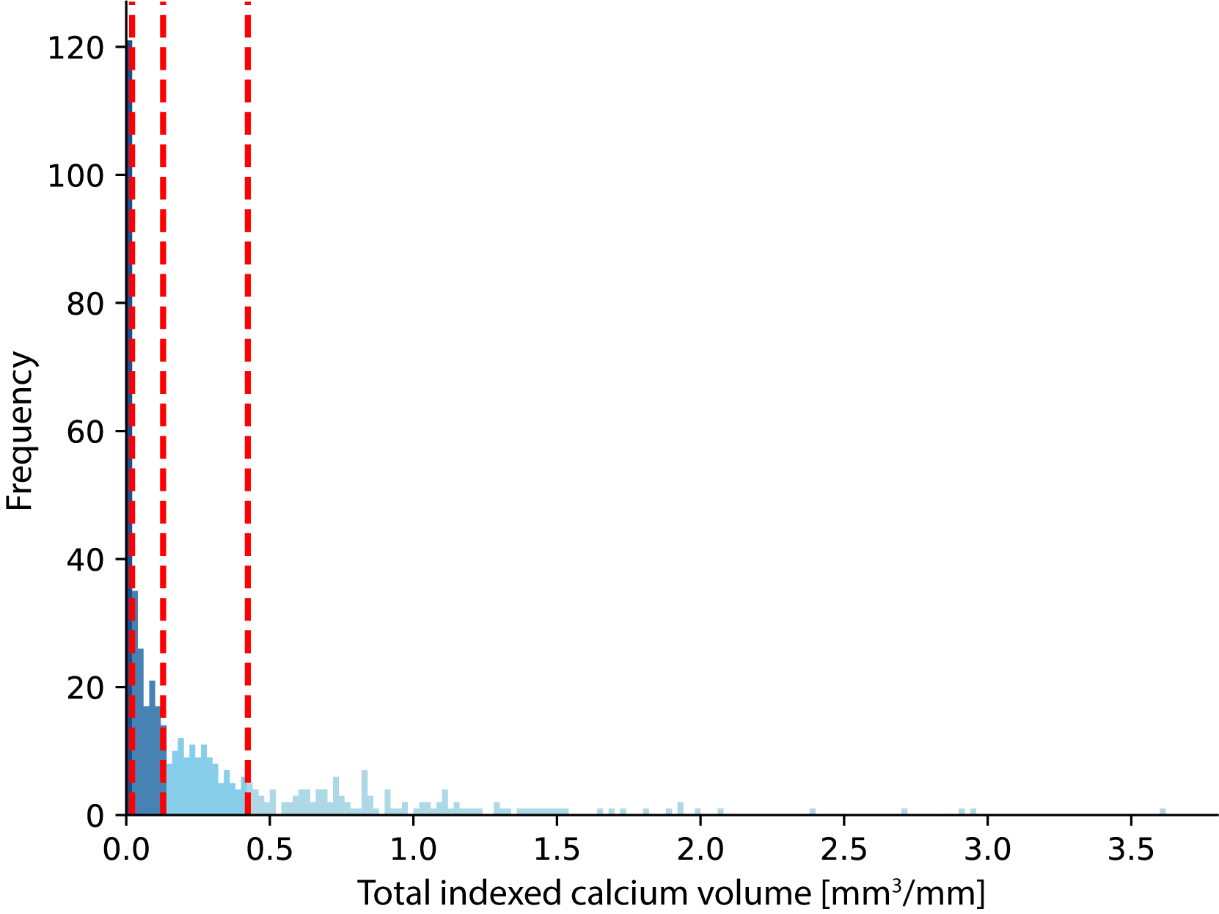


*Histogram of total indexed calcium volume. Red dashed lines indicate the cutoff values separating Q1-Q4.*

## Supplemental Figure 3 Individual lesion-level vulnerability features and total indexed calcium volume, stratified by lipid-lowering therapy

*
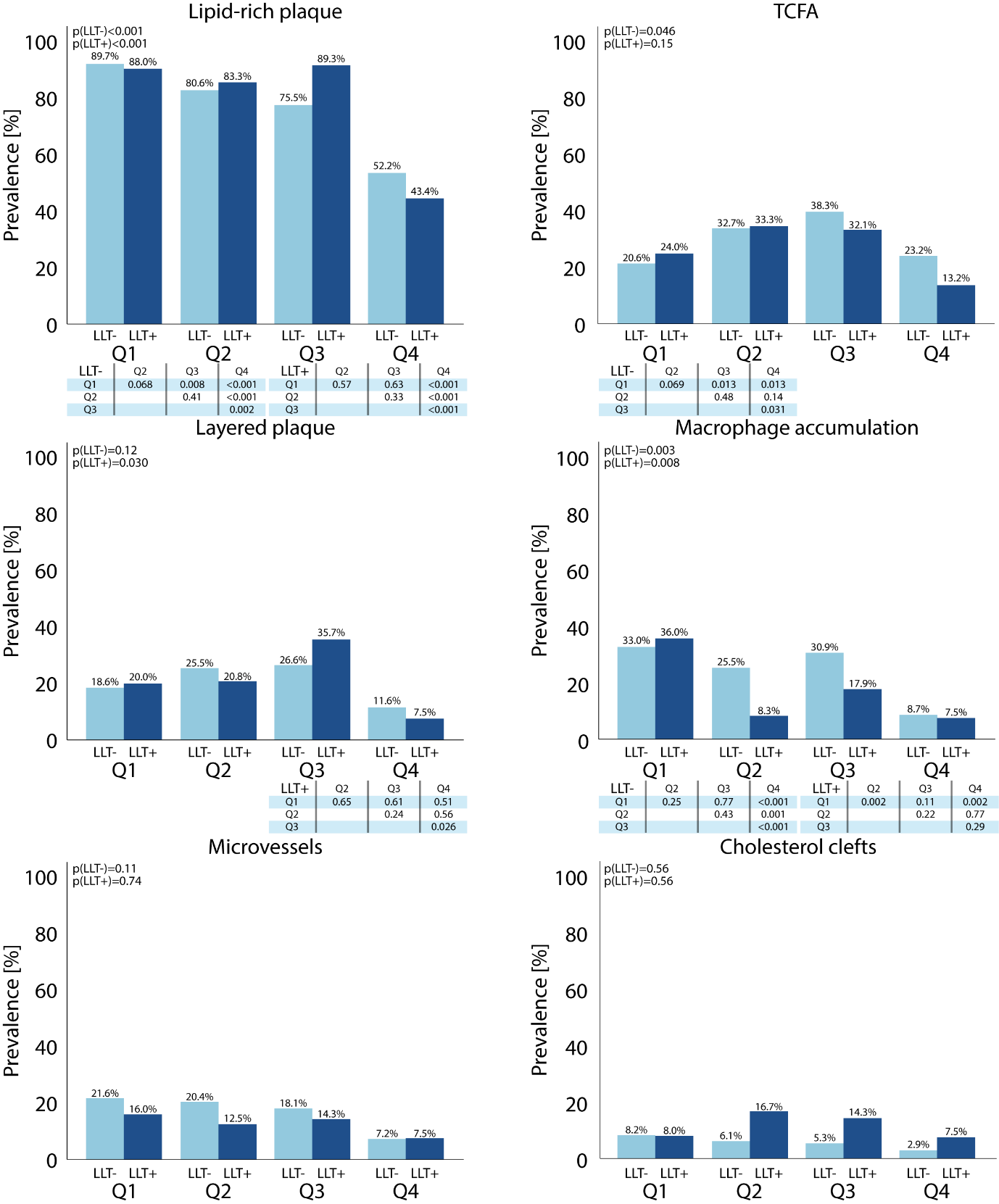
*

*Prevalence of individual plaque vulnerability features across indexed calcium volume quartiles, stratified by lipid-lowering therapy status (LLT- vs. LLT+). Pairwise comparison p-values between indexed calcium volume quartiles for individual plaque vulnerability feature, separately for LLT- and LLT+, are shown in the accompanying tables only when a significant across-group difference is present.*

*LLT, lipid-lowering therapy; TCFA, thin-cap fibroatheroma.*

## Supplemental Figure 4 Association between plaque vulnerability and total indexed calcium volume, stratified by lipid-lowering therapy

*
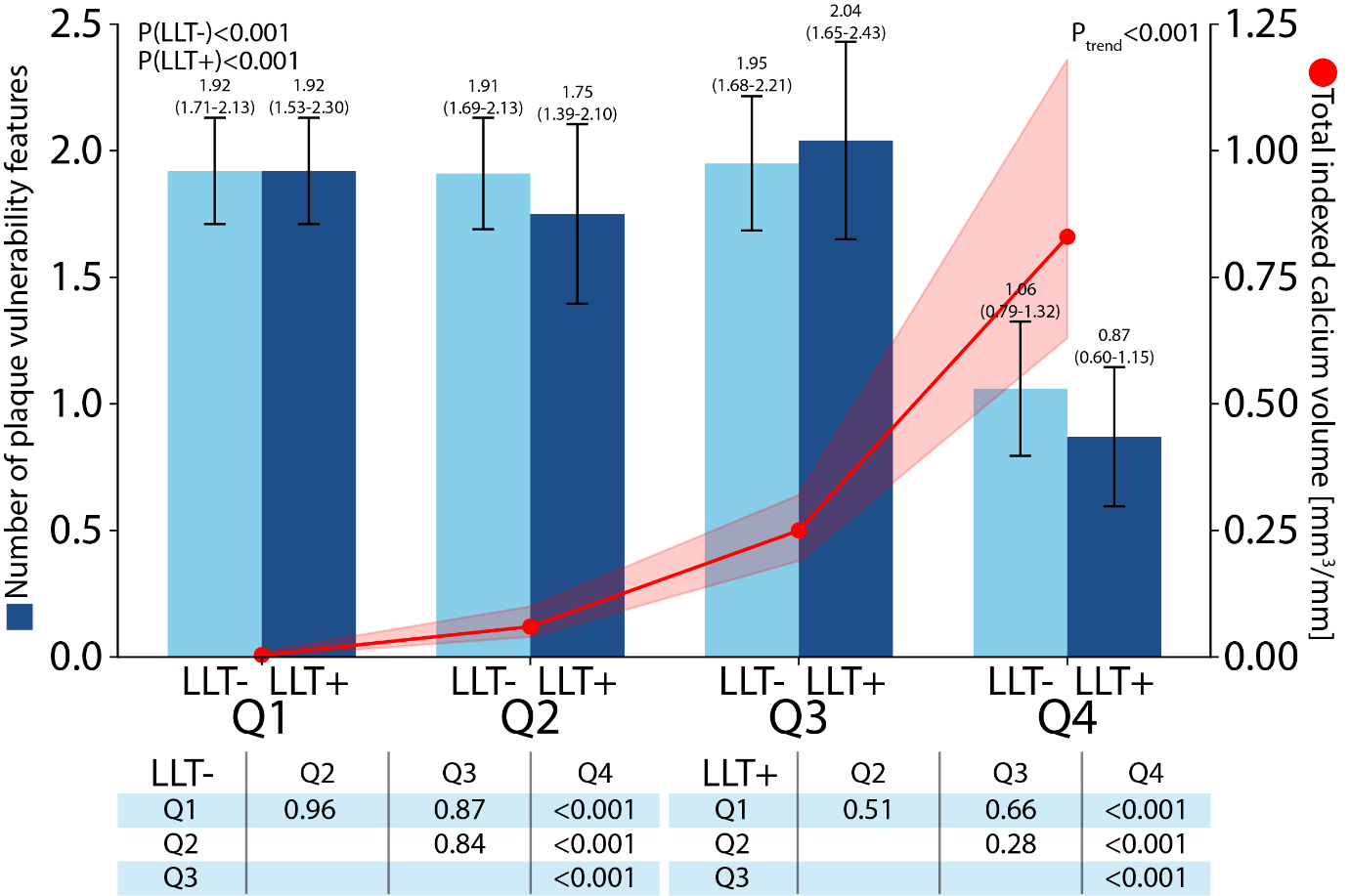
*

*Marginal mean (95% CI) number of plaque vulnerability features versus total calcium volume index per lesion stratified by lipid-lowering therapy (LLT- vs. LLT+), arranged left to right by increasing total index calcium volume quartiles. Pairwise comparison p-values between indexed calcium volume quartiles, separately for LLT- and LLT+, for number of plaque vulnerability features are shown in the accompanying table.*

*CI, confidence interval; LLT, lipid-lowering therapy.*

## Supplemental Figure 5 Individual lesion-level vulnerability features and calcium arc


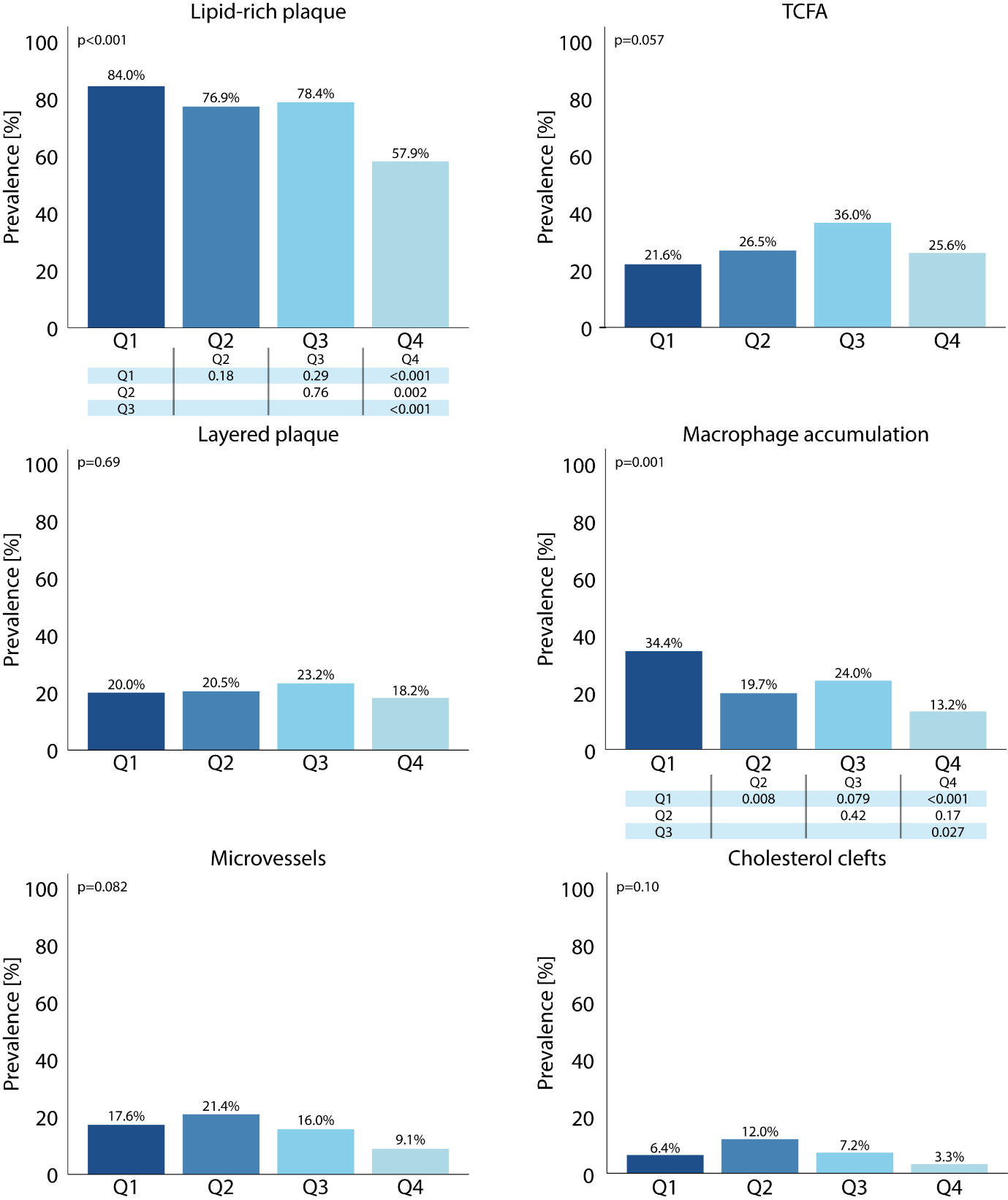


*Prevalence of individual plaque vulnerability features across calcium arc quartiles. Pairwise comparison p-values between calcium arc quartiles for individual plaque vulnerability feature are shown in the accompanying table only when a significant across-group difference is present.*

*TCFA, thin-cap fibroatheroma.*

## Supplemental Figure 6 Association between plaque vulnerability and calcium arc

*
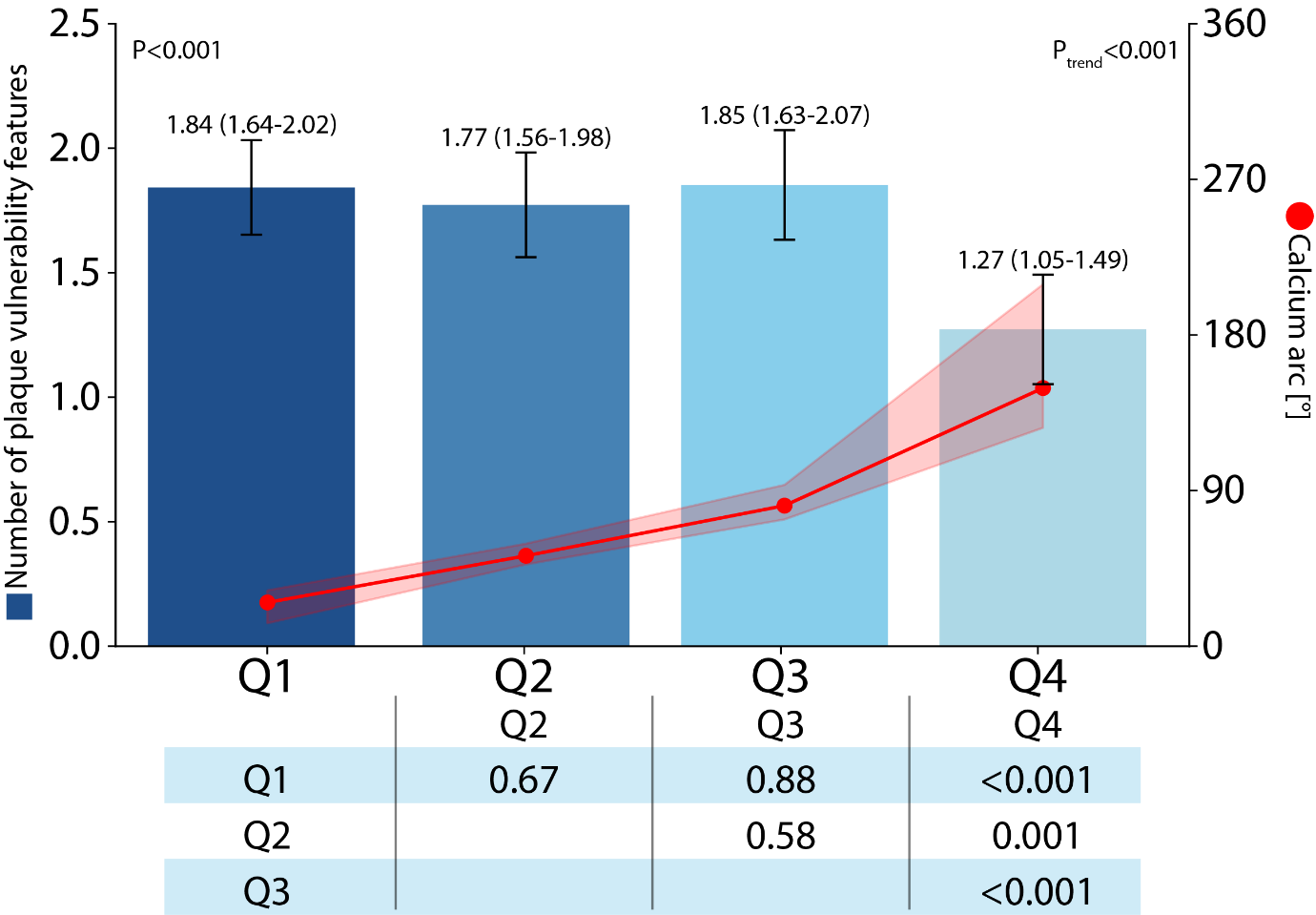
Marginal mean (95% CI) number of plaque vulnerability features versus calcium arc per lesion, arranged left to right by increasing calcium arc quartiles. Pairwise comparison p-values between calcium arc quartiles for number of plaque vulnerability features are shown in the accompanying table.*

*CI, confidence interval.*

## Supplemental Figure 7 Individual lesion‑level vulnerability features and total indexed calcium volume (calcium‑positive lesions only)

**
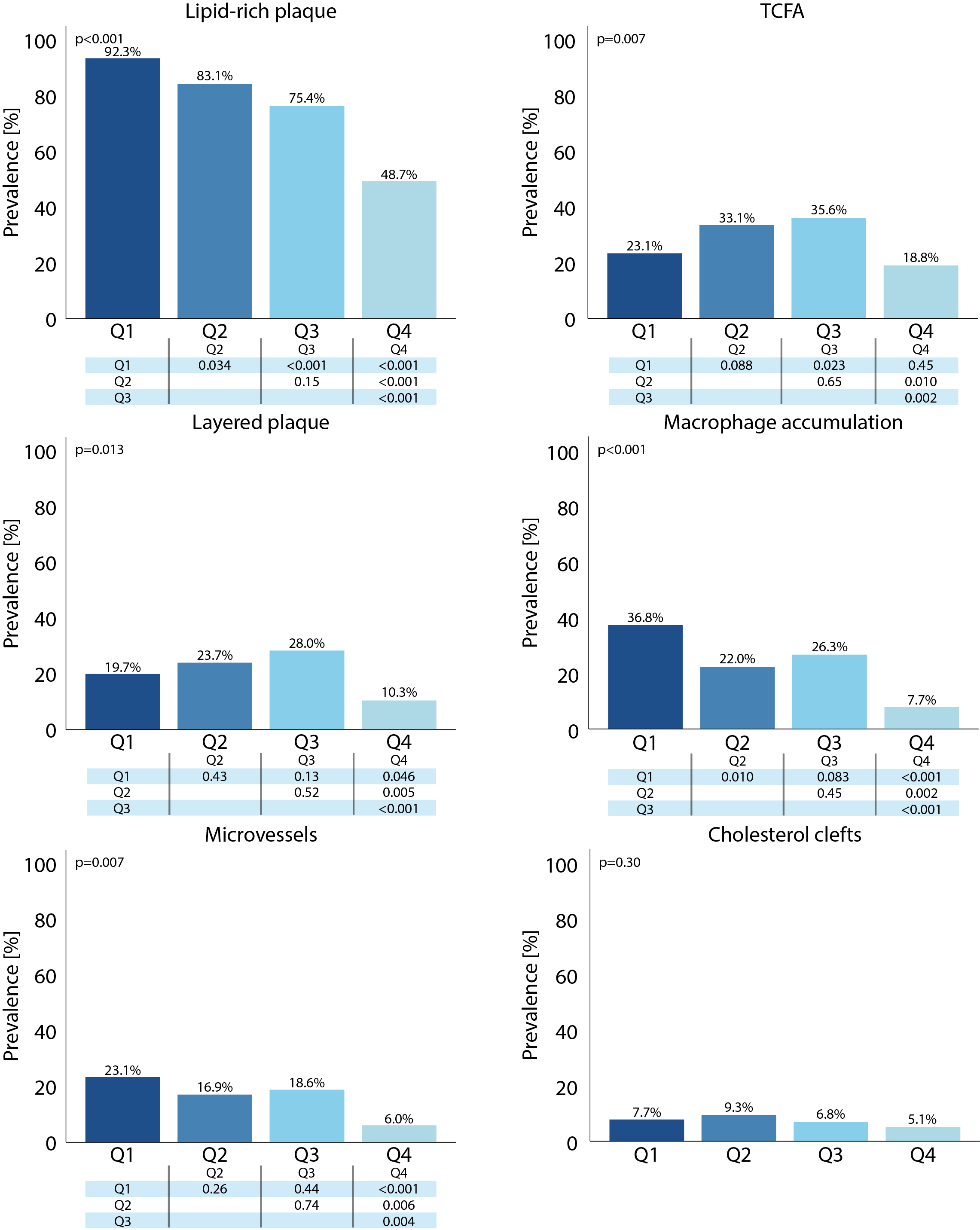
**

*Prevalence of individual plaque vulnerability features across indexed calcium volume quartiles. This analysis was performed using only calcium‑positive lesions, with quartiles recalculated accordingly (Q1: 117, Q2: 118, Q3: 118, Q4: 117). Pairwise comparison p-values between indexed calcium volume quartiles for individual plaque vulnerability feature are shown in the accompanying table only when a significant across-group difference is present.*

## Supplemental Figure 8 Association between plaque vulnerability and total indexed calcium volume (calcium‑positive lesions only)


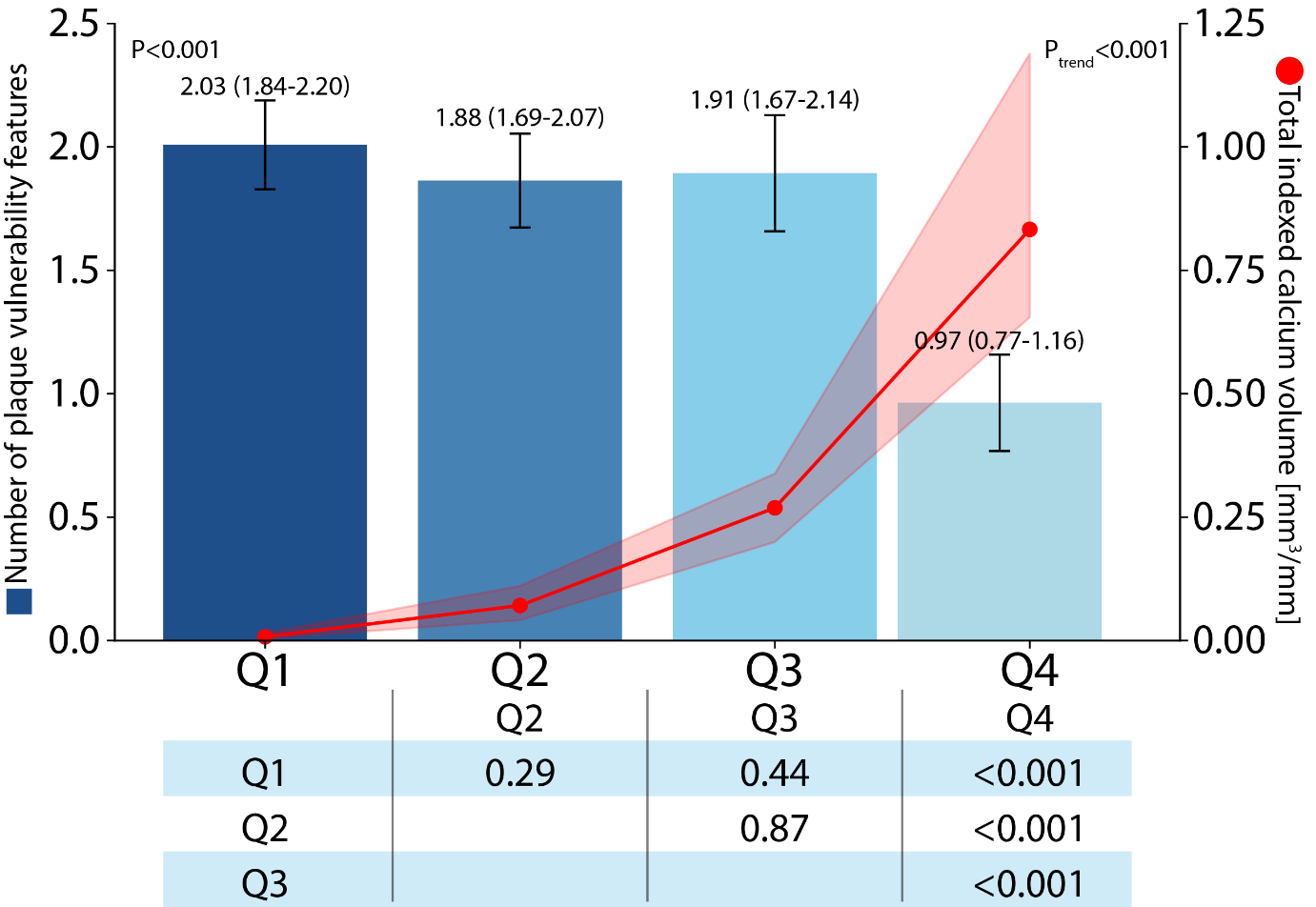


*Marginal mean (95% CI) number of plaque vulnerability features versus total calcium volume index per lesion, arranged left to right by increasing total index calcium volume quartiles. This analysis was performed using only calcium positive lesions, with quartiles recalculated accordingly (Q1: 117, Q2: 118, Q3: 118, Q4: 117). Pairwise comparison p-values between indexed calcium volume quartiles for number of plaque vulnerability features are shown in the accompanying table.*

*CI, confidence interval.*

# Supplemental Tables

## Supplemental Table 1 Lesion-level baseline characteristics per quartile

| **Variables** | **Q1 (n=122)** | **Q2 (n=122)** | **Q3 (n=122)** | **Q4 (n=122)** | **P-value** | **Q1 vs Q2** | **Q1 vs Q3** | **Q1 vs Q4** | **Q2 vs Q3** | **Q2 vs Q4** | **Q3 vs Q4** |
| --- | --- | --- | --- | --- | --- | --- | --- | --- | --- | --- | --- |
| Age (years) | 60±11 | 62±10 | 63±10 | 67±9 | 0.007 | 0.09 | 0.019 | <0.001 | 0.199 | 0.002 | 0.009 |
| Female sex | 22 (18.0%) | 17 (13.9%) | 28 (23.0%) | 21 (17.2%) | 0.31 |  |  |  |  |  |  |
| BMI (kg/m^2^) | 27.9±4.1 | 28.1±4.5 | 27.8±4.4 | 27.4±5.2 | 0.87 |  |  |  |  |  |  |
| Smoking |  |  |  |  | 0.59 |  |  |  |  |  |  |
| *Current smoking* | 32 (26.2%) | 46 (38.0%) | 34 (28.6%) | 33 (27.0%) |  |  |  |  |  |  |  |
| *Previous smoking* | 32 (26.2%) | 34 (28.1%) | 37 (31.1%) | 45 (36.9%) |  |  |  |  |  |  |  |
| Hypertension | 60 (49.2%) | 62 (50.8%) | 60 (49.2%) | 74 (60.7%) | 0.37 |  |  |  |  |  |  |
| Type 1 or 2 diabetes | 24 (19.7%) | 13 (10.7%) | 17 (13.9%) | 25 (20.5%) | 0.57 |  |  |  |  |  |  |
| Hypercholesterolemia | 40 (33.3%) | 45 (36.9%) | 44 (36.4%) | 52 (42.6%) | 0.74 |  |  |  |  |  |  |
| Family history of premature atherosclerosis | 26 (21.3%) | 42 (34.4%) | 35 (29.2%) | 40 (33.3%) | 0.13 |  |  |  |  |  |  |
| Previous MI | 19 (15.6%) | 15 (12.3%) | 17 (13.9%) | 25 (20.5%) | 0.60 |  |  |  |  |  |  |
| Previous PCI | 13 (10.7%) | 16 (13.1%) | 14 (11.5%) | 31 (25.4%) | 0.37 |  |  |  |  |  |  |
| Previous CVA | 1 (0.8%) | 1 (0.8%) | 2 (1.6%) | 5 (4.1%) | <0.001 | 0.97 | 0.26 | <0.001 | 0.54 | <0.001 | 0.017 |
| History of carotid artery disease | 5 (4.1%) | 3 (2.5%) | 3 (2.5%) | 3 (2.5%) | 0.70 |  |  |  |  |  |  |
| History of PAD | 5 (4.1%) | 1 (0.8%) | 4 (3.3%) | 9 (7.4%) | 0.06 |  |  |  |  |  |  |
| STEMI presentation | 68 (55.7%) | 68 (55.7%) | 56 (45.9%) | 56 (45.9%) | 0.31 |  |  |  |  |  |  |
| Cholesterol levels |  |  |  |  |  |  |  |  |  |  |  |
| *Total (mmol/L)* | 5.1±1.2 | 5.4±1.5 | 5.0±1.2 | 4.6±1.5 | 0.031 | 0.77 | 0.22 | 0.031 | 0.046 | 0.004 | 0.07 |
| *LDL (mmol/L)* | 3.3±1.1 | 3.4±1.3 | 3.1±1.2 | 2.6±1.2 | 0.019 | 0.69 | 0.13 | 0.007 | 0.13 | 0.002 | 0.016 |
| Triglyceride level (mmol/L) | 1.7 (1.0-2.6) | 1.7 (1.2-2.3) | 1.5 (1.0-2.1) | 1.6 (1.1-2.7) | 0.40 |  |  |  |  |  |  |
| eGFR (mL/min/1.73m^2^) | 79.9±17.3 | 81.5±19.3 | 80.4±18.7 | 75.7±21.3 | 0.36 |  |  |  |  |  |  |
| CRP (mg/L) | 3.0 (1.0-4.0) | 3.0 (1.8-6.0) | 2.8 (1.0-5.1) | 2.0 (1.0-7.0) | 0.056 |  |  |  |  |  |  |
| Leukocyte count (x10^9^/L) | 10.1±3.4 | 9.6±3.1 | 9.7±3.0 | 9.8±3.1 | 0.64 |  |  |  |  |  |  |
| Lipid-lowering therapy at presentation | 25 (20.5%) | 24 (19.7%) | 28 (23.0%) | 53 (43.4%) | 0.10 |  |  |  |  |  |  |

*Values are reported as counts (percentages), means ± standard deviations, or medians (interquartile range).*

*BMI, body mass index (calculated as weight in kilograms divided by height in meters squared); CRP, C-reactive protein; CVA, cerebrovascular accident; eGFR, estimated glomerular filtration rate; LDL, low-density lipoprotein; MaC, macrocalcification; MI, myocardial infarction; MiC, micro-calcification; PAD, peripheral artery disease; PCI, percutaneous coronary intervention; SC, spotty calcification; STEMI, ST-segment elevation myocardial infarction.*

## Supplemental Table 2 Binary comparison (Q1–Q3 vs Q4) of lesion-level vulnerability features

| **Presence** | **Q1-Q3 (n=366)** | **Q4 (n=122)** | **P-value** |
| --- | --- | --- | --- |
| Lipid-rich plaque | 304 (83.1%) | 59 (48.4%) | <0.001 |
| TCFA | 111 (30.3%) | 23 (18.9%) | 0.007 |
| Layered plaque | 88 (24.0%) | 12 (9.8%) | <0.001 |
| Macrophage accumulation | 102 (27.9%) | 10 (8.2%) | <0.001 |
| Microvessels | 69 (18.9%) | 9 (7.4%) | <0.001 |
| Cholesterol clefts | 29 (7.9%) | 6 (4.9%) | 0.21 |
| Mean number of plaque vulnerability features (95% CI) | 1.92 (1.81-2.03) | 0.98 (0.78-1.17) | <0.001 |

*CI, confidence interval; TCFA, thin-cap fibroatheroma.*

## Supplemental Table 3 Effect sizes for plaque vulnerability features across calcium volume quartiles

| **Presence** | **Q1-Q4** | **p-value** | **Q2-Q4** | **p-value** | **Q3-Q4** | **p-value** |
| --- | --- | --- | --- | --- | --- | --- |
| Lipid-rich plaque | 8.86  (4.50-17.42) | <0.001 | 4.61  (2.61-8.16) | <0.001 | 3.94  (2.28-6.79) | <0.001 |
| TCFA | 1.16  (0.61-2.22) | 0.65 | 2.10  (1.14-3.86) | 0.017 | 2.56  (1.42-4.60) | 0.002 |
| Layered plaque | 2.11  (1.00-4.44) | 0.050 | 2.97  (1.44-6.13) | 0.003 | 3.63  (1.74-7.60) | <0.001 |
| Macrophage accumulation | 5.73  (2.73-12.01) | <0.001 | 3.21  (1.51-6.83) | 0.002 | 4.38  (2.06-9.32) | <0.001 |
| Microvessels | 3.24  (1.42-7.39) | 0.005 | 2.92  (1.28-6.65) | 0.011 | 2.61  (1.11-6.12) | 0.027 |
| Cholesterol clefts | 1.74  (0.61-4.92) | 0.30 | 1.74  (0.61-4.92) | 0.30 | 1.51  (0.52-4.43) | 0.45 |
| Total number of plaque vulnerability features | 2.56  (2.00-3.34) | <0.001 | 2.46  (1.88-3.23) | <0.001 | 2.70  (2.00-3.63) | <0.001 |

*Values represent odds ratios with 95% confidence intervals from GEE models, with quartile 4 serving as the reference group.*

*CI, confidence interval; GEE, generalized estimating equations.*
